# Molecular Dipole Buffer Layer Enabling Compact Interfaces in Perovskite Solar Cells

**DOI:** 10.1021/acsenergylett.5c02004

**Published:** 2025-09-03

**Authors:** Danbi Kim, Chieh-Szu Huang, Weidong Xu, Lingxin Meng, Eui Dae Jung, Yoomi Ahn, Eunhye Yang, Yang Lu, Hongsuk Suh, Sung Heum Park, Samuel D. Stranks, Bo Ram Lee

**Affiliations:** † School of Advanced Materials Science and Engineering, 35017 Sungkyunkwan University (SKKU), Suwon 16419, Republic of Korea; ‡ Department of Chemical Engineering and Biotechnology, 2152 University of Cambridge, West Cambridge Site, Philippa Fawcett Drive, Cambridge CB3 0AS, United Kingdom; § Cavendish Laboratory, University of Cambridge, J. J. Thomson Avenue, Cambridge CB3 0HE, United Kingdom; ∥ Department of Chemistry and Chemistry Institute for Functional Materials, 34996 Pusan National University (PNU), Busan 46241, Republic of Korea; ⊥ Department of Physics, 34998 Pukyong National University, Busan 48513, Republic of Korea; # Department of Chemistry, 37580 National University of Singapore, Singapore 117543, Singapore

## Abstract

Despite advances in p-i-n perovskite solar cells, interfacial losses between the electron transport layer (ETL) and metal electrode remain a bottleneck for efficiency and stability. Bathocuproine (BCP), a common buffer layer, suffers from poor film uniformity, low electron mobility, and limited thermal stability. Here, we report BTI-N, a D–A–D-type small molecule featuring a benzo­[c]­[1,2,5]­thiadiazole core and polar N,N-dimethylamino groups. BTI-N exhibits favorable molecular packing and solubility, enabling compact, uniform films with efficient electron transport. The polar termini anchor Ag electrodes via Ag–N dipole formation, lowering the work function and improving band alignment and charge extraction. BTI-N also suppresses Ag and I ion diffusion, significantly enhancing thermal stability. We demonstrate broad compatibility across ETLs (C_60_, PCBM), electrodes (Ag, Au), and perovskites with bandgaps from 1.58 to 1.7 eV. This work provides a practical interface engineering strategy to replace BCP and realize high-performance, stable perovskite solar cells.

## Graphical abstract

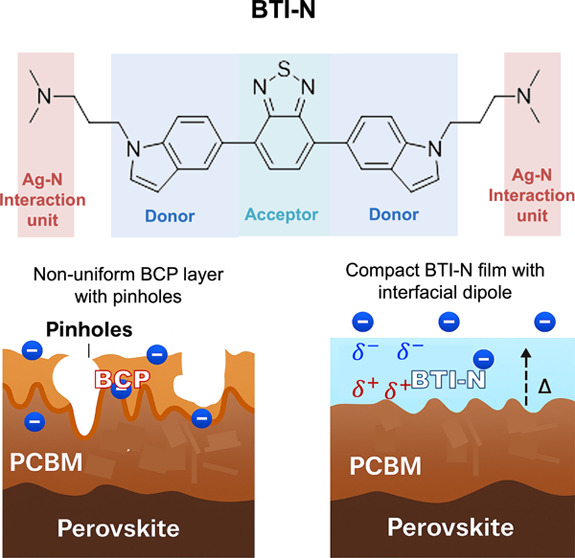

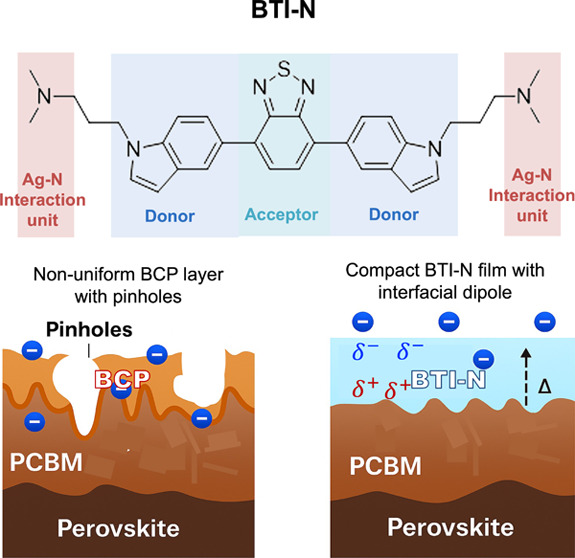

Halide perovskites have emerged as a promising next-generation photovoltaic technology due to their exceptional material characteristics, including a high absorption coefficient and long charge-carrier diffusion length.^1,2^ Among various device architectures, inverted (p–i–n) perovskite solar cells (PSCs) offer unique advantages such as compatibility with low-temperature processing and suitability for tandem integration via all-solution processes.^3,4^ Significant performance improvements in inverted PSCs have been achieved through interface engineering and defect passivation at perovskite/hole/electron transport layer (H/ETL) interfaces, which help suppress nonradiative recombination and minimize trap states.^5–9^ However, interface energy losses at the ETL-metal electrode interface remain relatively underexplored. Such losses, resulting from charge accumulation and low-resistance ″shunt pathways″ due to nonradiative recombination and energy-level mismatches between ETL materials (e.g., phenyl-C61-butyric acid methyl ester (PCBM) or C_60_) and metal electrodes, considerably reduce efficiency and stability.^7–14^ Addressing these critical interface issues requires further dedicated research to enable continued progress in device performance.

To address this, buffer layers are frequently introduced between the ETL and metal electrode to reduce energy barriers, facilitate charge extraction, and prevent interfacial degradation. Bathocuproine (BCP) is one of the most commonly employed buffer materials in high-performance inverted PSCs.^15,16^ Despite its widespread use, BCP suffers from several drawbacks, such as difficulty in forming uniform, pinhole-free films due to poor solubility and low surface energy, limited electron mobility, and poor thermal stability.^17–22^ Recent studies have pointed out that these morphological and electronic limitations are not merely due to processing defects, but rather stem from inherent characteristics of the BCP molecule itself, and are consistently observed in both PCBM- and C_60_-based fullerene ETLs.^10,16–18,20^ These issues increase series resistance, compromise charge extraction, and ultimately hinder device performance. Approaches such as side-chain functionalization^23–25^ and blending with additives^26–28^ have been explored to improve BCP’s film quality and energy alignment, but these strategies only partially address the interfacial limitations, and a fully satisfactory, molecule-level solution has yet to be realized. Perylene diimide (PDI)-based buffer layers have also been investigated to overcome the limitations of BCP.^29–34^ However, small-molecule PDIs^12,14,35–38^ tend to aggregate excessively, resulting in nonuniform films, while polymeric PDIs,^39–41^ though offering better coverage, introduce parasitic absorption and reduce overall device efficiency. Therefore, the development of a buffer material that ensures good film morphology, high charge extraction, and minimal optical loss remains a key challenge.

In this work, we report the development of a novel Donor–Acceptor–Donor (D–A–D)-type small molecule buffer layer, 3,3′-(benzo­[c]­[1,2,5]­thiadiazole-4,7-diylbis­(1H-indole-5,1-diyl))­bis­(N,N-dimethylpropan-1-amine) (BTI-N), designed to address the limitations of existing buffer materials. The molecular design incorporates a planar benzo­[c]­[1,2,5]­thiadiazole core flanked by indole units and terminated with dimethylamine groups, providing strong intermolecular interactions, good solubility, and strong chemical affinity to silver (Ag) electrodes via Ag–N dipole interactions. These features promote uniform film formation, efficient charge extraction, and enhanced interface stability.

Figure 1a shows the BTI-N structure; its synthesis via Suzuki coupling is shown in Scheme S1. The BTI-N material was characterized using ^1^H NMR and ^13^C NMR, as provided in the Supporting Information S1.1-S1.4. Initially, the electrochemical and optical properties of BTI-N were investigated, and the results are summarized in Table S1. UV–vis absorbance spectra in Figure S1 demonstrate absorbance at 422 nm in solution. Upon transitioning to the film state, a red shift of the peak to 438 nm is observed, indicating enhanced intermolecular interactions and improved molecular packing in the solid state.^42–44^ This enhanced packing originates from the fused rings and planar BT core stabilized by head-to-head hydrogen bonds, promoting the formation of homogeneous and compact thin films.^45–47^ Furthermore, the indole units in BTI-N facilitate strong intermolecular stacking, resulting in increased molecular planarity, improved film uniformity, and effective charge transport.^48,49^ The optical bandgap of BTI-N, determined from the absorption onset in the film state, is 2.39 eV. Additionally, cyclic voltammetry measurements (Figure S2) were used to estimate the highest occupied molecular orbital and lowest unoccupied molecular orbital energy levels, which were determined to be −5.22 eV and −3.22 eV, respectively.

**Figure 1:**
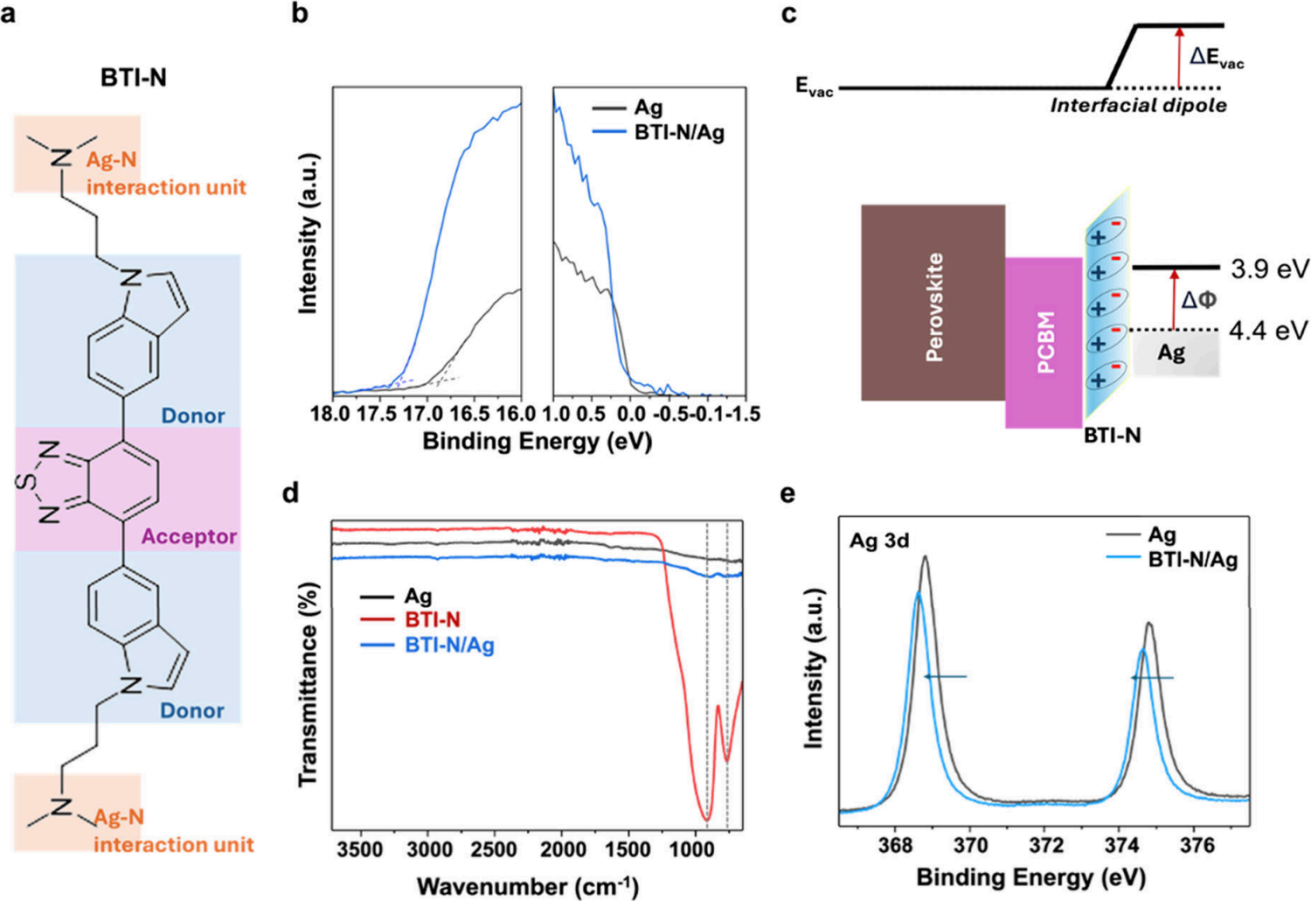
(a) Molecular structure of BTI-N, (b) UPS spectra of Ag with and without the BTI-N coating, (c) schematic energy level diagram illustrating the work function (WF) modification of Ag by the BTI-N buffer layer used in PSCs, (d) FTIR spectra of Ag, BTI-N, and BTI-N/Ag films, and (e) XPS spectra of Ag and BTI-N/Ag films.

Next, the interfacial interactions between BTI-N and thermally evaporated Ag electrodes were examined. The polar *N*, *N*-dimethyl amino pendant groups of BTI-N provide a chelation site for thermal evaporated Ag.

The strong dipole interaction from Ag–N bonding reduces the work function of Ag, which aligns the interface charge distribution, enhancing charge mobility and extraction.^29,50^ To clearly confirm the impact of the Ag–N dipole interaction on the energy band alignment, ultraviolet photoelectron spectroscopy (UPS) analysis was conducted (Figure 1b,c). Figure 1b shows the UPS spectra of bare Ag electrodes compared with those coated by the BTI-N layer. The results clearly indicate that the BTI-N coating significantly reduces the WF of the Ag electrode from 4.4 eV (bare Ag) to 3.9 eV (BTI-N/Ag), as illustrated in Figure 1c. This notable WF reduction induces an upward shift in the electrode energy levels, resulting in enhanced built-in potential and improved charge extraction efficiency in devices. Fourier transform infrared (FTIR) spectroscopy was then conducted to compare pristine pure Ag films, BTI-N films and Ag-deposited BTI-N films (Figure 1d). The FTIR spectrum of pristine BTI-N exhibited two distinct peaks: one at 918 cm^–1^ corresponding to the N–H wagging vibration peak and another at 765 cm^–1^ attributed to aromatic ring deformation vibrations.^51,52^ Upon deposition of Ag onto BTI-N, significant changes were observed. The N–H wagging vibration shifted from 918 cm^–1^ to 899 cm^–1^. Consequently, the N–H bond is weakened, resulting in a lower vibrational frequency. This suggests that nitrogen atoms in the amine groups of BTI-N anchor Ag ions, reducing the electron density around the nitrogen.^53,54^ Additionally, the intensity of the 765 cm^–1^ peak decreased substantially, reflecting structural and electronic changes in the aromatic system likely caused by electron density redistribution upon Ag interaction.

To further confirm the interaction between BTI-N and Ag, X-ray Photoelectron Spectroscopy (XPS) analysis was performed. The XPS spectra of the Ag 3d regions for pristine and Ag-deposited BTI-N films are shown in Figure 1e. In the Ag 3d region, a decrease in binding energy and a shift in peak position toward the negative direction were observed. These changes further validate the interaction between BTI-N and Ag, accompanied by a change in the electronic state of Ag.^50^ In addition, this Ag–N coordination promotes more uniform nucleation and denser Ag film formation during thermal evaporation, improving the top electrode coverage and interfacial contact quality.^25^

Careful atomic force microscopy (AFM) was employed to investigate the surface morphologies and film uniformity of BCP and BTI-N buffer layers on the PCBM ETL layer (Figure 2). The PCBM film exhibited a hilly and slightly textured surface (Figure 2a, d), providing a baseline. When BCP was deposited, the resulting surface showed sharp and irregular features with pronounced local spikes and valleys (Figure 2b, e), reflecting the challenge of forming a uniform film due to BCP’s limited solubility and weaker interaction with PCBM.

**Figure 2:**
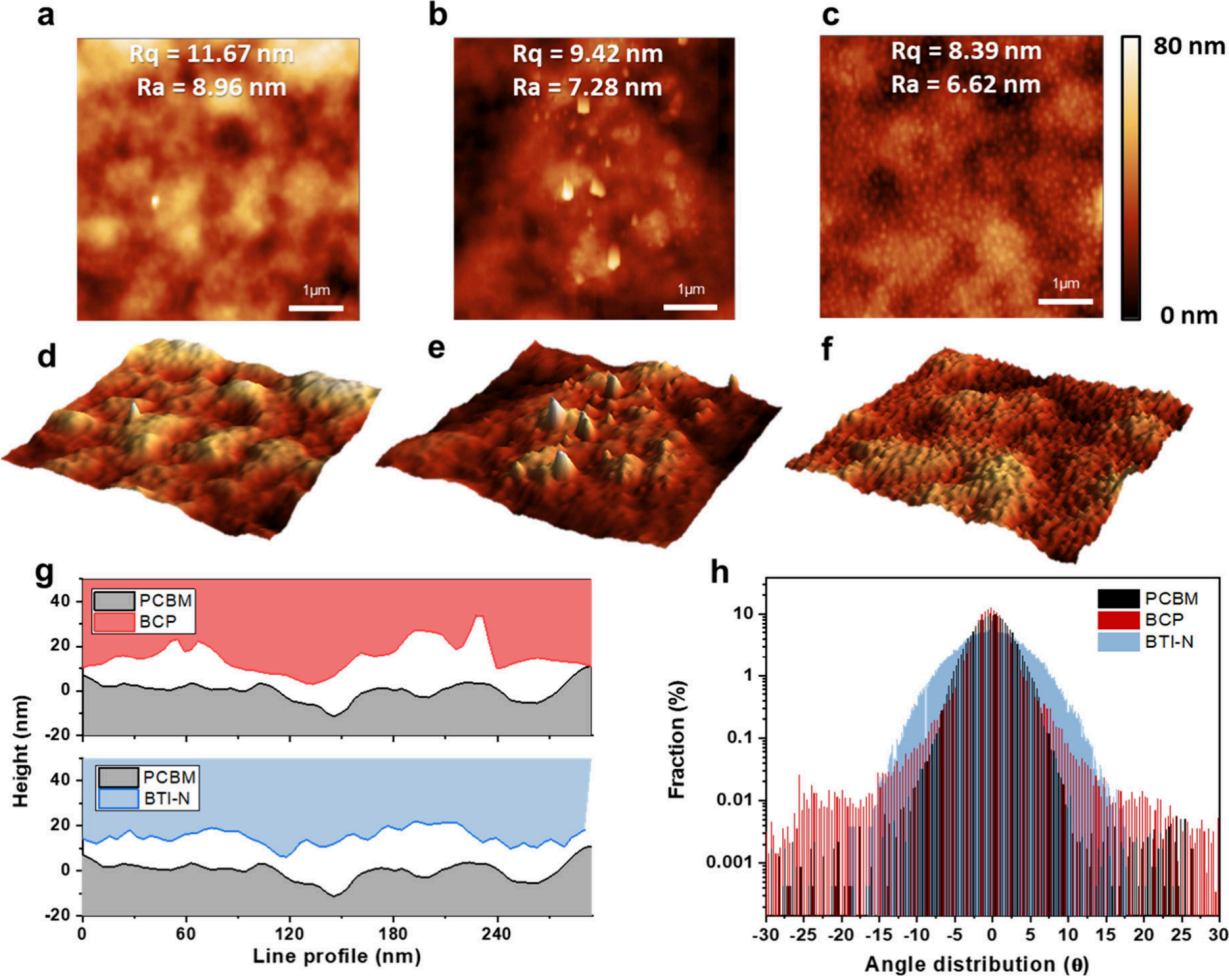
(a)-(f) 2D and 3D AFM image of PCBM (a,d), BCP/PCBM (b,e), and BTI-N/PCBM film (c,f), (g) height line profiles and (h) statistical angle distribution of PCBM, BCP/PCBM, and BTI-N/PCBM films.

To further investigate the solubility differences, we compared BCP and BTI-N solutions at concentrations of 0.5 mg/mL and 5 mg/mL in Figure S3. In the case of BCP, undissolved particles were clearly visible in the 5 mg/mL solution, confirming its poor solubility at higher concentrations. By contrast, BTI-N remained fully dissolved even at 5 mg/mL, resulting in a clear solution. This high solubility is consistent with the morphological observations: the BTI-N-modified surface exhibited densely packed, small, and rounded features, forming a compact and uniform film on top of PCBM (Figure 2c, f). This smooth morphology is attributed to BTI-N’s excellent solubility and strong intermolecular stacking.

Surface roughness was evaluated by root-mean-square (R_q_) and averaged roughness (R_a_) values: PCBM-only showed 11.67 and 8.96 nm, BCP/PCBM 9.42 and 7.28 nm, and BTI-N/PCBM the lowest at 8.39 and 6.62 nm (Table S2). These results indicate that BTI-N provides superior surface coverage and reduced interfacial roughness. Height profiles (Figure 2g) further support this, showing large fluctuations for BCP/PCBM, whereas BTI-N/PCBM shows smaller and more consistent variations. The height line profiles (Figure 2g) quantitatively support these observations from the height and depth line-profiling of the data in Figure 2a-f.^55^ To compare the PCBM surface with those of BCP/PCBM and BTI-N/PCBM, Figure 2g presents the PCBM profile (black) as the baseline, while the BCP/PCBM (red) and BTI-N/PCBM (blue) profiles are shown inverted above for direct comparison. The BCP/PCBM film exhibits large height fluctuations, indicative of a rough and nonuniform surface. By contrast, the BTI-N/PCBM film shows smaller and more consistent variations, confirming its uniformity and smoother coverage. Statistical angle distribution analysis (Figure 2h) further corroborates these results, where the angle distribution refers to a measurement that roughly indicates the degree of curvature from an ideal flat surface.^55,56^ The BCP-modified surface shows a broader distribution with a noticeable fraction above 10° and even beyond 15°, indicating abrupt local height changes.

Conversely, the BTI-N-modified surface displays a narrow, centered distribution with no high-angle components, demonstrating its superior flatness and more homogeneous film formation. This compact, rounded-feature morphology enables more conformal and continuous Ag film growth during top electrode deposition, as the smooth underlying surface reduces interfacial voids and promotes uniform nucleation. This morphological difference is attributed to the intrinsic molecular characteristics of BTI-N. Its benzo­[c]­[1,2,5]­thiadiazole core and planar indole units with a D–A–D structure promote π–π stacking and ordered molecular packing,^45–49^ while the polar N,N-dimethylamino side groups enhance solubility and interfacial affinity with the PCBM layer.^12,31,37^ These combined features enable uniform spreading and dense film formation even at low concentrations, in contrast to the small and less interactive structure of BCP, which often results in incomplete or inhomogeneous coverage.

To evaluate the impact of BTI-N on device performance, inverted PSCs with a structure of indium tin oxide­(ITO)/2-(3,6-dimethoxy-9*H*-carbazol-9-yl)­ethyl]­phosphonic acid (MeO-2PACz) (HTL)/perovskite/PCBM (ETL)/Ag were fabricated, comparing PCBM-only, BCP/PCBM, and BTI-N/PCBM configurations. The perovskite is nominally comprised of FA_0.92_MA_0.08_Pb­(Br_0.92_I_0.08_)_3_ (FA= CH­(NH_2_)_2_, MA = CH_3_NH_3_) with CsI 5%. Scanning electron microscopy (SEM) images shown in Figure S4 illustrate the layer thicknesses of MeO-2PACz (∼5 nm), perovskite (∼500 nm), PCBM (∼30 nm), BTI-N or BCP (∼10 nm), and Ag (∼100 nm). The current density–voltage (*J–V*) characteristics of the champion devices (from 50 devices) are presented in Figure 3a, with statistical distributions in Figure 3b and Figure S5, and summarized in Table 1.

**Figure 3:**
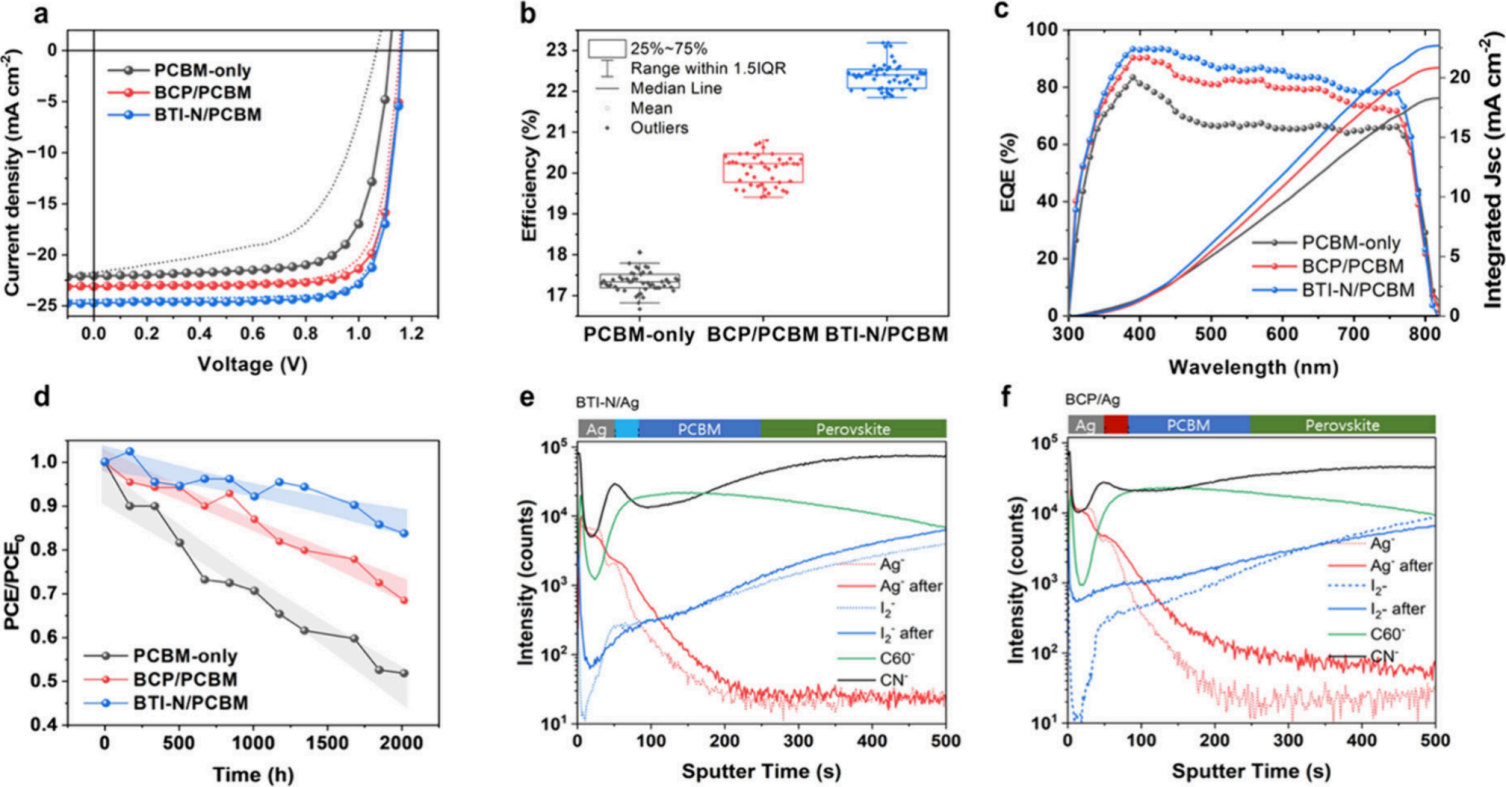
(a) *J–V* characteristics of devices with PCBM-only, BCP/PCBM, and BTI-N/PCBM layers, (b) statistical distribution of PCEs for PCBM-only, BCP/PCBM, and BTI-N/PCBM-based devices, (c) EQE spectra of devices with PCBM-only, BCP/PCBM, and BTI-N/PCBM layers, (d) operational stability of devices with the three buffer layer configurations under continuous illumination, (e) ToF-SIMS spectra of BTI-N/Ag, and (f) ToF-SIMS spectra of BCP/Ag after thermal stress at 120 °C in ambient conditions for 24 h.

**Table 1:** *J–V* Characteristics of PSC under AM1.5G (100 mW/cm^2^)­^a^

	*J*_*sc*_ (mA/cm^2^)	*V*_*oc*_ (V)	FF	PCE (%)
PCBM-only	22.06 (21.02 ± 0.28)	1.12 (1.06 ± 0.07)	0.73 (0.70 ± 0.07)	18.07 (17.36 ± 0.28)
BCP/PCBM	23.11 (22.17 ± 0.22)	1.16 (1.14 ± 0.10)	0.80 (0.77 ± 0.05)	21.45 (20.23 ± 0.53)
BTI-N/PCBM	24.38 (23.97 ± 0.18)	1.16 (1.15 ± 0.07)	0.82(0.80 ± 0.04)	23.19 (22.39 ± 0.32)

a The average values are stated in parentheses and are obtained from 50 individual devices.

The BTI-N/PCBM device exhibited the highest efficiency of 23.19% (average PCE of 22.39 ± 0.32%) with a short-circuit current density (*J*_*sc*_) of 24.38 mA/cm^2^, an open-circuit voltage (*V*_*oc*_) of 1.16 V, and a FF of 0.82. In comparison, the PCBM-only device achieved an efficiency of 18.07% (average PCE of 17.36 ± 0.28%), with *J*_*sc*_ of 22.06 mA/cm^2^, *V*_*oc*_ of 1.12 V, and FF of 0.73, while the BCP/PCBM device demonstrated an efficiency of 21.45% (average PCE of 20.23 ± 0.53%), with *J*_*sc*_ of 23.11 mA/cm^2^, *V*_*oc*_ of 1.16 V, and FF of 0.80. EQE spectra (Figure 3c) confirmed the highest integrated *J_sc_* for BTI-N/PCBM (22.75 mA/cm^2^), followed by BCP (20.90 mA/cm^2^) and PCBM-only (18.91 mA/cm^2^).

The difference between EQE-derived and *J*_*sc*_ values was within ≈5% for all devices, indicating reliable measurement consistency. Notably, BTI-N/PCBM devices showed enhanced EQE across the full visible spectrum, suggesting suppressed nonradiative recombination and more efficient charge collection. The superior device performance observed with BTI-N is not only due to higher efficiency but is also supported by consistent improvements across all photovoltaic parameters—*V*_*oc*_, *J*_*sc*_, and FF—compared to the PCBM-only device. The application of both BCP and BTI-N buffer layers led to enhancements in all parameters relative to the PCBM-only configuration, with the BTI-N device showing particularly greater improvements in *J*_*sc*_ and FF than BCP. These improvements are attributed to BTI-N’s better interfacial contact, higher film uniformity, and effective energy-level alignment, which facilitate more efficient charge extraction and suppressed recombination. To further verify the general applicability of BTI-N across different ETL and perovskite compositions, we additionally fabricated devices using a wide-bandgap (WBG) perovskite (Eg ≈ 1.7 eV) and C60 as the ETL, comparing C_60_–BCP and C_60_–BTI-N configurations. As shown in Figures S6, S7 and Table S3 the BTI-N-based devices outperformed both evaporated and spin-coated BCP devices in terms of *J*_*sc*_ and PCE. Figure S8 and Table S4 further show that our device exhibits competitive PCE among reported 1.67–1.70 eV systems. The average PCE of the C_60_–BTI-N devices reached 19.43 ± 0.42%, compared to 18.34 ± 0.73% and 17.80 ± 0.68% for the evaporated and spin-coated BCP controls, respectively. These results confirm that the beneficial interfacial effects of BTI-N, including improved contact, reduced recombination, and efficient charge extraction, are consistently observed even in wide-bandgap perovskite systems and with C_60_-based ETLs.

To further explore effectiveness of BTI-N with high-WF electrodes, we fabricated additional wide-bandgap devices using gold (Au) as the top contact. As shown in Figure S9 and Table S5, the device without any interfacial layer (C_60_–Au) exhibited poor performance, with an average PCE of 11.73  ± 0.64% and a low FF of 0.57. In contrast, the insertion of BTI-N between C_60_ and Au led to a remarkable increase in device performance, yielding an average PCE of 18.57  ± 0.33% and an FF of 0.81. Notable enhancements were also observed in *J*_*sc*_ and *V*_*oc*_. This significant improvement is attributed to the dipole-induced work function tuning effect of BTI-N at the Au interface. Kelvin probe measurements confirmed a reduction in the work function of Au by approximately 0.54 eV upon BTI-N modification (Figure S10), validating that BTI-N can effectively align the energy levels and facilitate charge extraction even with high-WF electrodes. These results highlight the versatility of BTI-N as a universal cathode interlayer across various ETL materials and metal electrodes. Figure 3d shows the stability performance of the three devices over 2000 h under continuous 1-sun illumination in a nitrogen-filled glovebox at ambient temperature, without encapsulation. *J–V* measurements were taken every 168 h. The PCBM-only device showed the most severe degradation, retaining only 51% of its initial PCE. The BCP/PCBM device retained 68%, while the BTI-N/PCBM device maintained 83%, demonstrating significantly improved long-term stability.

To further assess interfacial degradation under thermal stress, full device structures were thermally aged at 120 °C for 24 h in ambient air. As shown in Figure S11, BCP/Ag devices showed pronounced yellowing of the perovskite layer and visible deformation of the electrode, indicating accelerated degradation. In contrast, BTI-N/Ag devices exhibited minimal discoloration and preserved electrode morphology. These visual differences were corroborated by cross-sectional scanning electron microscopy (SEM) and energy dispersive X-ray spectroscopy (EDS) mapping, which revealed extensive Ag and I^–^ diffusion across multiple layers in BCP-based devices. In comparison, BTI-N suppressed such migration, maintaining sharp elemental interfaces.

Time-of-flight secondary ion mass spectrometry (ToF-SIMS) depth profiling was conducted on BCP/Ag and BTI-N/Ag structures (with ∼20 nm Ag), before and after aging at 120 °C for 24 h in ambient air (Figure 3e, f). Initially, both devices showed similar Ag distributions. However, after thermal stress, significant Ag diffusion into the perovskite layer was observed in the BCP/Ag device, while the BTI-N/Ag retained a sharp interface with negligible Ag migration. This is attributed to strong Ag–N dipole interactions and compact morphology of BTI-N, which effectively block metal diffusion. Similarly, ToF-SIMS profiles of I^–^ showed that BCP/Ag allowed I^–^ penetration into the Ag layer, while BTI-N/Ag suppressed I^–^ migration, with minimal signal changes after thermal stress. These results confirm the superior chemical robustness of BTI-N, which not only enhances initial performance but also mitigates interfacial degradation by trapping migrating Ag and I^–^ ions. This reduces trap formation and AgI-related recombination losses.^57^ By contrast, PCBM-only and BCP/PCBM devices lack such protective features, leading to faster degradation and poorer operational stability.

To gain insights into the origin of enhanced photovoltaic performance, we investigated the charge transport and recombination processes in PSCs through the various measurements. First, the light dependence of FF (Figure S12) showed that BTI-N/PCBM devices maintained high FF values even under low light, indicating reduced interfacial recombination and improved shunt resistance. By contrast, other two devices exhibited lower FF values, indicating higher interface defect density and less efficient charge transport. This observation was further corroborated by analyzing the light dependence of *V*_*oc*_, as presented in Figure S13. The light dependence of *V*_*oc*_ was used to calculate the ideality factor *n*_*id*_ based on the following equation:

$$n_{id}=\frac{q}{k_{B}T}\times \frac{dV_{oc}}{d(\ln\left(I\right))}$$

where *n*_*id*_ is the ideality factor, *q* is the elementary charge, *k*_*B*_ is the Boltzmann constant, *T* is the absolute tempearture, and *I* is the light intensity.

The parameter *n*_*id*_, which typically ranges between 1 and 2, indicates the extent of trap-assisted charge recombination within the device.^58,59^ *n*_*id*_ were calculated as 1.41 ± 0.03 for BTI-N-based devices, 1.55 ± 0.02 for BCP-based devices, and 1.65 ± 0.04 for devices without a buffer layer. Among these, the BTI-N-based devices exhibited the lowest *n*_*id*_, indicating minimized interface recombination losses and enhanced charge transport. We further analyzed interfacial charge carrier transport kinetics using electrochemical impedance spectroscopy (EIS) and space-charge-limited current (SCLC) measurements with detailed resistance values and electron mobilities summarized in Table S6. Nyquist plots (Figure S14) revealed that BTI-N/PCBM devices had the lowest series resistance (R_1_) and highest recombination resistance (R_2_), indicating efficient charge extraction and suppressed surface energy losses, likely due to Ag–N dipole interactions.^50,60–62^

SCLC measurements were performed to investigate charge trap density and mobility. Electron-only devices (ITO/ZnO/perovskite/PCBM or BCP or BTI-N/Ag) were tested via dark *J–V* (Figure S15). The trap density *N*_*trap*_ was calculated using the following equation:

$$N_{trap}=\frac{2\varepsilon_{o}\varepsilon_{r}V_{TFL}}{qL^{2}}$$

where *N*_*trap*_ is the trap density, *ε*_*o*_ is the vacuum permittivity, *ε*_*r*_ is the relative dielectric constant, *V*_*TFL*_ is the trap-filled limit voltage, *q* is the elementary charge, and *L* is thickness of the perovskite film.^63^

From these measurements, we determined threshold values of 0.76, 0.62, and 0.52 V for devices without a buffer layer, with BCP, and with BTI-N, respectively. The corresponding *N_trap_* were calculated 3.22 × 10^15^ cm^–3^, 1.32 × 10^15^ cm^–3^ and 7.29 × 10^14^ cm^–3^. Electron mobilities were measured to be 1.79 × 10^–6^ cm^2^V^–1^s^–1^ (PCBM-only), 1.02 × 10^–5^ cm^2^V^–1^s^–1^ (BCP), and 5.12 × 10^–5^ cm^2^V^–1^s^–1^ (BTI-N), as summarized in Table S5. Dark *J–V* measurements were also conducted to evaluate leakage current characteristics (Figure S16). The dark current densities were 1.57 × 10^–3^ mA/cm^2^ for PCBM-only, 2.97 × 10^–4^ mA/cm^2^ for BCP, and 1.74 × 10^–4^ mA/cm^2^ for BTI-N. The significantly lower dark current density in BTI-N-based devices indicates suppressed leakage pathways and reduced recombination, contributing to the observed improvements in *J*_*sc*_ and FF. These results confirm that the BTI-N buffer layer effectively reduces defect density, enhances charge extraction, and promotes better device performance through the formation of a uniform and compact interface with the PCBM layer.

To investigate the impact of these buffer layers on microscale morphology and optoelectronic properties distributions within completed devices, wide-field microscopic PL mapping measurement was conducted using a 405 nm continuous-wave laser (170 mW/cm^2^), as described in the Supporting Information. Figure 4a–c and 4d–f show photoluminescence (PL) distributions for PCBM-only, BCP/PCBM, and BTI-N/PCBM devices under open-circuit (OC) and short-circuit (SC) conditions, respectively. At OC, the PCBM-only device exhibited the lowest PL intensity, indicating significant nonradiative recombination, consistent with its lower *V_oc_*. By contrast, BCP and BTI-N devices showed stronger PL at OC, suggesting reduced nonradiative recombination and improved *V*_*oc*_. Under SC, the BTI-N device showed the most efficient PL quenching, reflecting enhanced charge extraction and reduced carrier accumulation—correlating well with the higher *J*_*sc*_ and FF. It has been well established that high PL of a PSC at OC indicates reduced charge nonradiative recombination in the device, where more photogenerated charges contribute to *V*_*oc*_; insufficient quenching of PL in a PSC at SC is indicative of enhanced charge accumulation at SC, indicative of photogenerated charges not being extracted, which leads to reduced *J*_*sc*_.^64–66^ These results suggest that introducing buffer layers at the PCBM/Ag interface is necessary to reduce nonradiative recombination at OC and improve charge extraction at SC, indicating a better ohmic contact on the cathode side of the PSC, with BTI-N demonstrating the best effect of those explored here. This conclusion aligns with the discussion of the UPS results and the SCLC results, where the Ag work function is modified and overall electron mobility in the device is improved by employing BTI-N. Moreover, the device with BTI-N shows the best PL uniformity both at OC and SC (see Figure 4g,h for the histogram plot), indicating more uniform charge distribution and extraction in this device. This uniformity is attributed to the compact and continuous BTI-N film on top of PCBM, as supported by AFM analysis.

**Figure 4:**
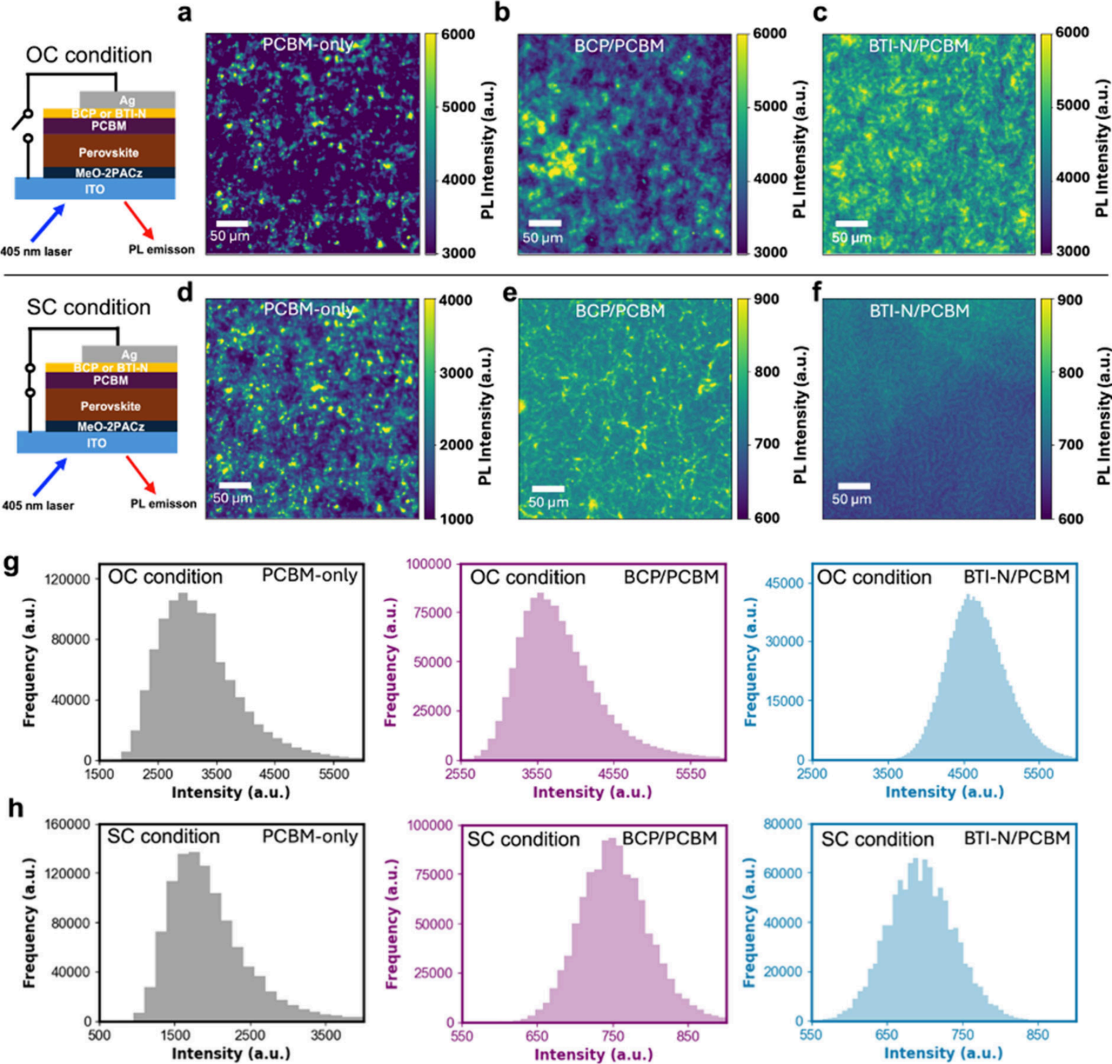
(a–f) Wide-field microscopic photoluminescence (PL) mapping of perovskite solar cells incorporating PCBM-only, BCP/PCBM, and BTI-N/PCBM layers under open-circuit (OC) conditions (a–c) and short-circuit (SC) conditions (d–f). (g, h) Histogram plots of PL intensity under OC (g) and SC (h) conditions, respectively. All PL measurements were performed using a 405 nm continuous-wave laser with an excitation intensity of 170 mW cm^–2^.

This study demonstrates that a D-A-D-type small molecule BTI-N is a superior buffer layer for inverted PSCs compared to BCP, owing to the intrinsic high charge transport, high film uniformity, and Ag-anchoring capability. These features enhance charge extraction, transport efficiency, and device stability. The BTI-N layer facilitated strong Ag–N dipole interactions, reducing the work function of Ag, and formed compact, homogeneous thin films, optimizing charge transfer, suppressing recombination, and enhancing charge mobility even under low-light conditions. As a result, device efficiency improved from 18.07% (PCBM-only) to 23.19% (BTI-N/PCBM), with 83% PCE retained after 2000 h under continuous 1-sun illumination in a N_2_ glovebox with periodic *J–V* measurements, versus 68% for BCP and 51% without a buffer. ToF-SIMS confirmed minimal Ag and I^–^ diffusion, highlighting BTI-N’s superior barrier properties. These results highlight BTI-N as a highly promising alternative to BCP for advancing PSC efficiency and stability.

## Supplementary Material



## Notes

Detailed experimental procedures and additional data (UV–visible absorbance spectra; Cyclic voltammetry curves; R_q_ and R_a_ values measured by AFM; SEM; Statistical distribution of *J*_*sc*_, *V*_*oc*_, FF; light-intensity dependence of FF; Light-intensity dependence of *V*_*oc*_; Nyquist plots from EIS measurements; SCLC on electron-only device; The dark *J*–*V* characteristic curves) (PDF)
