# Supplementary material for: Molecular Dipole Buffer Layer Enabling Compact Interfaces in Perovskite Solar Cells
Source: ACS Energy Lett. 2025 Sep 3;10(9):4712–21. doi: 10.1021/acsenergylett.5c02004 (PMC12442069; doi:10.1021/acsenergylett.5c02004)
Supplement: Supplementary file 1 [file nz5c02004_si_001.pdf]

Supporting Information

Molecular Dipole Buffer Layer Enabling Compact Interfaces in Perovskite Solar Cells

Danbi Kim,[1,2,3] Chieh-Szu Huang,[2] Weidong Xu,[2,3] Lingxin Meng,[4] Eui Dae Jung,[1] Yoomi Ahn,[5,6] Eunhye Yang,[5] Yang Lu,[2,3] Hongsuk Suh,[4] Sung Heum Park,[*5] Samuel D. Stranks[*2,3] and Bo Ram Lee[*1]

[1]School of Advanced Materials Science and Engineering, Sungkyunkwan University (SKKU), Suwon 16419, Republic of Korea.

[2]Department of Chemical Engineering and Biotechnology, University of Cambridge, West Cambridge Site, Philippa Fawcett Drive, Cambridge CB3 0AS, United Kingdom.

[3]Cavendish Laboratory, University of Cambridge, J. J. Thomson Avenue, Cambridge CB3 0HE, United Kingdom.

[4]Department of Chemistry and Chemistry Institute for Functional Materials, Pusan National University (PNU), Busan, 46241 Republic of Korea.

[5]Department of Physics, Pukyong National University, Busan 48513, Republic of Korea.

[6]Department of Chemistry, National University of Singapore, Singapore 117543, Singapore.

* Corresponding authors

E-mail: spark@pknu.ac.kr(Sung Heum Park), sds65@cam.ac.uk(Samuel D. Stranks), brlee@skku.edu(Bo Ram Lee).

Experimental Section

Synthesis of BTI-N

Synthesis of 6-Bromo-1-(3-bromopropyl)-1H-indole (Compound a)

The reagents and starting materials were purchased from commercial suppliers (Aldrich, TCI Korea, or Thermo Fisher Scientific) and used without further purification. To a stirred solution of 6-bromo-1H-indole (4.9 g, 25.0 mmol) in DMF (40.0 mL) under an argon atmosphere were added K_2CO_3 (23.2 g, 167.7 mmol), 18-crown-6 (0.35 g, 1.7 mmol), and 1,3-dibromopropane (29.8 mL, 167.8 mmol). The reaction mixture was stirred at 30 °C overnight. After completion, the mixture was poured into water (100.0 mL) and extracted with ethyl acetate (EA) (2 × 150.0 mL). The combined organic layers were washed with water (2 × 50.0 mL), dried over Na_2SO_4, and concentrated under reduced pressure. The crude product was purified by column chromatography, yielding compound a as a yellow oil (4.6 g, 43.2%).

^{1}H NMR (400 MHz, $CDCl_3$): δ (ppm) 7.75 (d, J = 1.8 Hz, 1H), 7.33–7.20 (m, 2H), 7.14 (d, J = 3.1 Hz, 1H), 6.44 (dd, J = 3.2, 0.7 Hz, 1H), 4.30 (t, J = 6.4 Hz, 2H), 3.27 (t, J = 6.1 Hz, 2H), 2.32 (d, J = 6.3 Hz, 2H). ^{13}C NMR (100 MHz, $CDCl_3$): δ (ppm) 134.57, 130.39, 129.16, 124.55, 123.58, 112.89, 110.75, 101.24, 44.12, 32.66, 30.27. HRMS (m/z, EI^+) calcd for $C_{11}H_{11}Br_2N$: 315.9336; found: 315.9332.

Synthesis of 3-(6-Bromo-1H-indol-1-yl)-N,N-dimethylpropan-1-amine (Compound b)

To a solution of compound a (2.0 g, 6.3 mmol) in THF (50.0 mL) under an argon atmosphere was added isopropylamine (5.4 mL, 63.0 mmol). The reaction mixture was stirred at 40 °C overnight. After concentration under reduced pressure, the residue was extracted with chloroform (2 × 150.0 mL). The combined organic layers were washed with water (2 × 100.0 mL), dried over Na_2SO_4, and concentrated under reduced pressure. The crude material was purified by column chromatography, yielding compound b as a yellow oil (0.99 g, 56.4%).

^{1}H NMR (400 MHz, $CDCl_3$): δ (ppm) 7.74 (d, J = 1.8 Hz, 1H), 7.32–7.21 (m, 2H), 7.11 (d, J = 3.1 Hz, 1H), 6.42 (d, J = 3.1 Hz, 1H), 4.17 (t, J = 6.8 Hz, 2H), 2.124–2.16 (m, 8H), 1.98–1.92 (m, 2H). ^{13}C NMR (100 MHz, $CDCl_3$): δ (ppm) 134.75, 130.22, 129.18, 124.20, 123.35, 112.55, 110.94, 100.63, 56.23, 45.44, 44.08, 28.19. HRMS (m/z, EI^+) calcd for $C_{13}H_{17}BrN_2$: 281.0653; found: 281.0652.

Synthesis of N,N-Dimethyl-3-(6-(4,4,5,5-tetramethyl-1,3,2-dioxaborolan-2-yl)-1H-indol-1-yl)propan-1-amine (Compound c)

Compound b (2.8 g, 10.0 mmol) was dissolved in anhydrous THF (50.0 mL) and cooled to −78 °C

under an argon atmosphere. To this solution, n-butyllithium (2.5 M in hexane, 5.0 mL, 12.0 mmol) was added dropwise and stirred at −78 °C for 1 hour. Then, 2-isopropoxy-4,4,5,5-tetramethyl-1,3,2-dioxaborolane (5.3 mL, 15.0 mmol) was added in one portion. The mixture was stirred at room temperature overnight, quenched with ice water (50.0 mL), and extracted with methylene chloride (MC) (3 × 100.0 mL). The combined organic layers were washed with water (3 × 100.0 mL), dried over Na_2SO_4, and concentrated under reduced pressure. The crude product was purified by column chromatography, yielding compound c as a white solid (1.0 g, 46.3%).

^{1}H NMR (400 MHz, $CDCl_3$): δ (ppm) 8.16 (s, 1H), 7.64 (d, J = 8.2 Hz, 1H), 7.36 (d, J = 8.4 Hz, 1H), 7.10 (d, J = 3.1 Hz, 1H), 6.50 (d, J = 3.1 Hz, 1H), 4.19 (t, J = 6.9 Hz, 2H), 2.22 (d, J = 6.7 Hz, 2H), 1.97 (d, J = 6.9 Hz, 2H), 1.36 (s, 12H), 1.24 (d, J = 1.1 Hz, 6H). ^{13}C NMR (100 MHz, $CDCl_3$): δ (ppm) 138.01, 128.92, 128.35, 128.12, 127.50, 108.84, 101.80, 83.39, 82.85, 56.38, 45.37, 43.99, 28.17, 26.23, 25.43, 24.91, 24.83, 24.64, 13.90. HRMS (m/z, EI^+) calcd for $C_{19}H_{28}BN_2O_2$:329.2420; found: 329.2408.

Synthesis of 3,3'-(Benzo[c][1,2,5]thiadiazole-4,7-diylbis(1H-indole-5,1-diyl))bis(N,N-dimethylpropan-1-amine)

A mixture of 4,7-dibromobenzo[c][1,2,5]thiadiazole (0.3 g, 1.0 mmol), compound c (1.3 g, 4.0 mmol), and $Pd(PPh_3)_4$ (35.0 mg, 0.03 mmol) in degassed toluene (15.0 mL) and K_2CO_3 solution (2 M, 10.0 mL) was stirred at 110 °C for 72 hours. After cooling to room temperature, the mixture was extracted with methylene chloride (MC) (3 × 100.0 mL). The combined organic layers were washed with water (3 × 100.0 mL), dried over Na_2SO_4, and concentrated under reduced pressure. The crude product was purified by column chromatography, yielding the final product as a yellow solid (0.39 g, 72.6%).

^{1}H NMR (400 MHz, $CDCl_3$): δ (ppm) 8.23 (s, 2H), 7.87–7.79 (m, 4H), 7.53 (d, J = 8.5 Hz, 2H), 7.18 (d, J = 3.1 Hz, 2H), 6.61 (dd, J = 3.1, 0.8 Hz, 2H), 4.26 (t, J = 6.9 Hz, 4H), 2.32–2.28 (m, 16H), 2.03 (d, J = 7.1 Hz, 4H). ^{13}C NMR (100 MHz, $CDCl_3$): δ (ppm) 153.6, 148.3, 136.7, 129.8, 126.5, 123.4, 120.8, 118.9, 110.3, 102.5, 56.8, 45.9, 44.6, 28.8. HRMS (m/z, EI^+) calcd for $C_{32}H_{36}N_6S$: 537.2810; found: 537.2792.

Materials

Methylammonium bromide (MABr, 99%) and formamidinium iodide (FAI, 99%) were purchased from Greatcell Solar. Cesium iodide (CsI) was obtained from Sigma-Aldrich. Lead iodide (PbI_2, 99.99%) and lead bromide ($PbBr_2$, >98.0%) were procured from TCI. MeO-2PACz was also purchased from TCI. PCBM was supplied by EMNI, and bathocuproine (BCP) was sourced from Sigma-Aldrich. All

solvents, including chlorobenzene (CB), chloroform (CF), ethanol, N,N-dimethylformamide (DMF), and dimethyl sulfoxide (DMSO), were purchased from Sigma-Aldrich.

Devices Fabrication

First, the ITO glass substrate was thoroughly cleaned using detergent, followed by sequential 15-minute sonication treatments in water, acetone, and IPA to ensure a pristine surface. The substrate was then dried overnight in an oven and subjected to 15 minutes of plasma treatment to enhance hydrophilicity.

The MeO-2PACz was dissolved in anhydrous ethanol at a concentration of 0.5 mg/mL and sonicated for 15 minutes to ensure complete dissolution. Within a glovebox, the solution was deposited onto the substrate and allowed to sit for 5 seconds before undergoing a spin-coating process at 3000 rpm for 30 seconds. The coated substrate was subsequently annealed at 100 °C for 10 minutes.

The mixed perovskite solution was prepared by combining individual perovskite solutions in the appropriate volume ratios. The CsI solution was prepared by dissolving 1.5 M CsI in DMSO, while the $FAPbI_3$ solution was prepared by dissolving 1.4 M $FAPbI_3$ with 10% excess PbI_2 in a 4:1 DMF/DMSO solvent mixture. Similarly, the $MAPbBr_3$ solution was prepared by dissolving 1.4 M $MAPbBr_3$ in a 4:1 DMF/DMSO mixture. These three solutions were combined to prepare the CsI (5%) and $FA_{0.83}MA_{0.17}Pb(I_{0.83}Br_{0.17})_3$ perovskite solution. The WBG perovskite solution of 0.75 M $Cs_{0.25}FA_{0.75}Pb(I_{0.73}Br_{0.27})_3$ was prepared by dissolving CsI, FAI, PbI_2, and PbBr, in a 4:1 (vol:vol) mixture of DMF and DMSO. The mixed solution was immediately filtered through a 0.22 μm PTFE filter.

A two-step spin-coating method was employed to deposit the perovskite layer on top of the MeO-2PACz layer. The spin-coating process included an initial spin at 500 rpm for 7 seconds, followed by a second spin at 5000 rpm for 25 seconds. During the second spin, 300 μL of chlorobenzene (CB) was dropped onto the substrate 7 seconds before the spin ended. After spin coating, each substrate was promptly transferred to a hot plate and heated at 100 °C for 30 minutes.

A PCBM solution (20 mg/mL in CB) was spin-coated onto the perovskite film at 2000 rpm for 40 seconds, or 20 nm of C_{60} was deposited. Subsequently, either BTI-N or BCP dissolved in ethanol (0.5 mg/mL) was spin-coated onto the PCBM layer at 5000 rpm for 30 seconds or 7 nm of BCP was deposited. Finally, a 100 nm thick silver electrode was deposited as the cathode under a vacuum pressure of 5×10^{-5} Torr.

Measurements and Characterizations

The ^{1}H-NMR and ^{13}C-NMR spectra were recorded using a Bruker Ascend 400 MHz spectrometer. UV-vis absorption spectra were measured with a Varian 5E UV/VIS/NIR spectrophotometer. Cyclic voltammetry (CV) measurements of the target compounds were conducted using a Wona-WPG100 system in a three-electrode configuration at room temperature with a scan rate of 80 mV/s. The reference electrode used was $Ag/AgNO_3$, and the counter electrode was platinum (Pt). The electrolyte consisted

of a 0.1 M solution of tetrabutylammonium perchlorate dissolved in acetonitrile. The energy level of the Ag/$AgNO_3$ reference electrode, calibrated against the Fc/Fc^+ redox system, was determined to be 4.4 eV below the vacuum level. The effective work function was measured using a Kelvin probe (KP Technology). The current density–voltage (*J*–*V*) characteristics of the devices, SCLC and dark *J*-*V* were measured under an N_2 atmosphere using a Keithley 2400 source meter. J–V curves were obtained under simulated AM 1.5G solar illumination with an intensity of 100 mW/cm^2. The incident photon-to-current efficiency (IPCE) spectra were recorded using a Keithley 2400 source meter under monochromatic light irradiation from a xenon lamp. Electrical impedance spectroscopy (EIS) measurements and Mott-Schottky analyses were performed using an Ivium compactStat.h electrochemical analyzer. Nyquist plots and the equivalent circuit with a fitted series resistance (R_1), a recombination resistance (R_2) at a bias of – 1.0 V under dark condition. Additionally, the constant phase element (CPE) represents a capacitive element. X-ray photoelectron spectroscopy (XPS) measurements were carried out with an AXIS SUPRA system (KRATOS Analytical Ltd.). Fourier-transform infrared (FTIR) spectra were obtained using a JASCO FT-4100 spectrometer. To elucidate the interaction between BTI-N and metal electrodes, we fabricated Ag-deposited BTI-N films with a thinner Ag layer (~3 nm) on BTI-N. Ultraviolet photoelectron spectroscopy (UPS) data were measured on a Theta probe (Thermo) system. Atomic force microscopy (AFM) analysis was performed using a high-resolution MFP-3D AFM system (Asylum Research/Oxford Instruments) operated in tapping mode, employing aluminum-coated silicon probes (Tap-150Al-G, BudgetSensors). For each buffer layer condition, five independent samples were measured, with three to five areas scanned per sample to ensure statistical consistency and representative evaluation of surface morphology. Time-of-flight secondary ion mass spectrometry were performed using a Hitachi H-7500 and ION ToF-SIMS 5. ToF-SIMS depth profiling was performed on devices with BCP/Ag and BTI-N/Ag structures before and after heating at 120 °C for 24 h in ambient conditions (Figure 3e, f), using a thin Ag layer (~20 nm).-Wide-field microscopy was conducted using a Photon etc. IMA system, equipped with a 20× magnification Olympus objective lens (NA = 0.8). To correct for optical aberrations, the system was calibrated with a reference sample, allowing us to determine the post-processing parameters needed to correct image distortions. Chromatic aberrations were minimized by calibrating the z-position of the sample for each collected wavelength. A continuous wave 405 nm laser with intensity of 170 mw/cm^2 served as the excitation source, filtered through a high-quality 405 nm Semrock dichroic mirror to separate the photoluminescence signal from the excitation. The emitted light from the sample was directed onto a Hamamatsu CMOS camera with a 2048×2048 pixel array. During the measurement, the encapsulated device was switched to open-circuit or short-circuit by connected to a Keithley 2450.

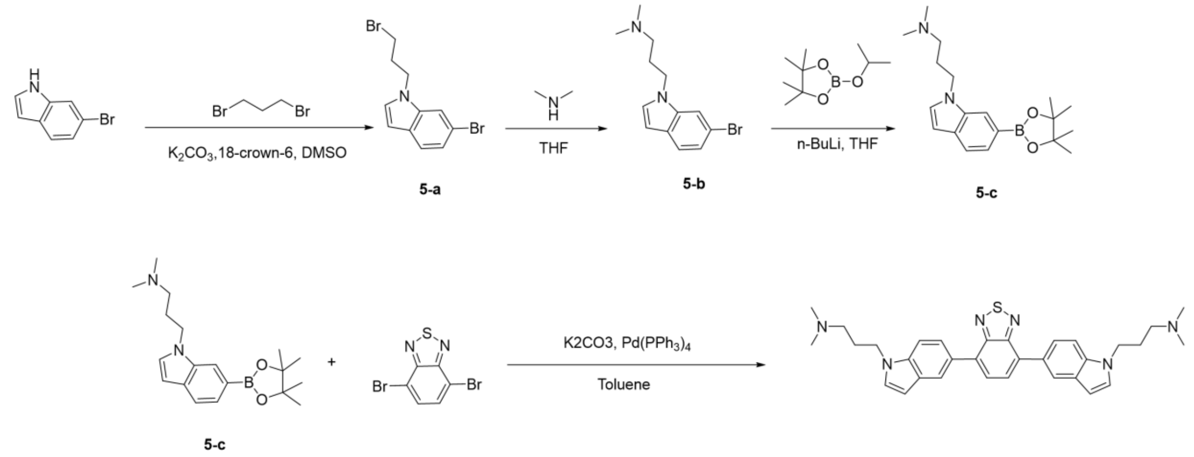

Scheme S1. Synthetic routes of BTI-N

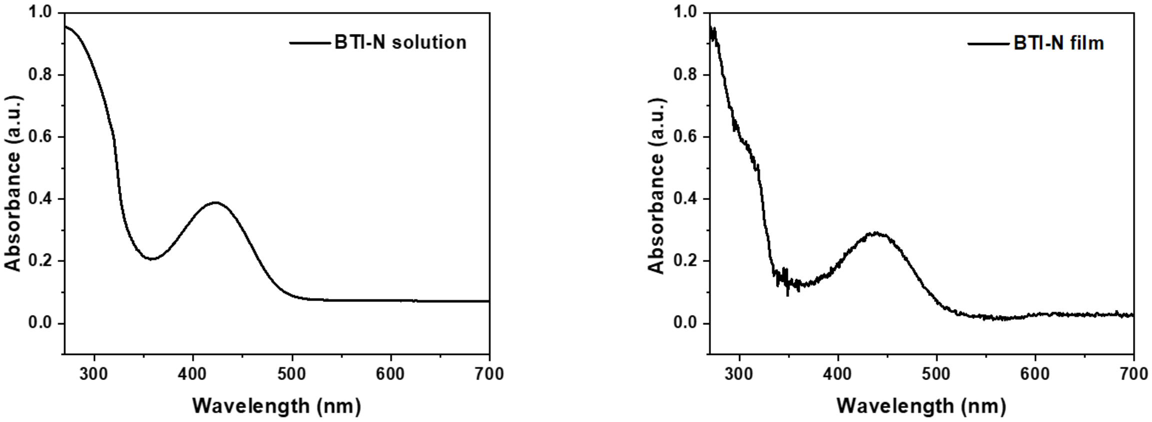

Figure S1. UV-visible absorbance spectra of BTI-N in the methylene chloride solution and fil m state.

Table S1. Optical and Electrochemical properties of BTI-N.

Sol λ_{max} (nm)	film λ_{max} (nm)	film λ_{oneset} (nm)	Eg^{opt} (eV)	HOMO[a] (eV)	LUMO [b] (eV)	Eg [elc,c] (V)
422	438	518	2.39	-5.12	-3.22	1.90

[a] Calculated from oxidation potentials,[b] Calculated from reduction potentials,[c] Calculated from E_{ox} and E_{red}.

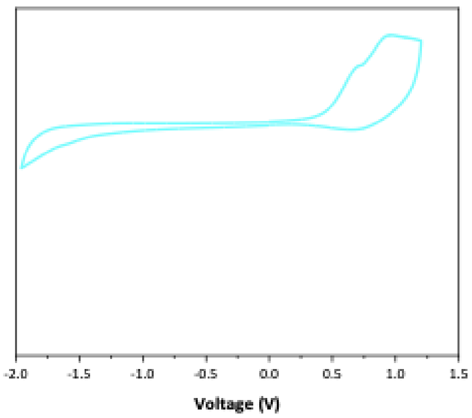

Figure S2. Cyclic voltammetry curves of BTI-N in 0.1 M TBAF in acetonitrile solution at a sca n rate of 100 mV/s at room temperature (vs an Ag quasi-reference electrode).

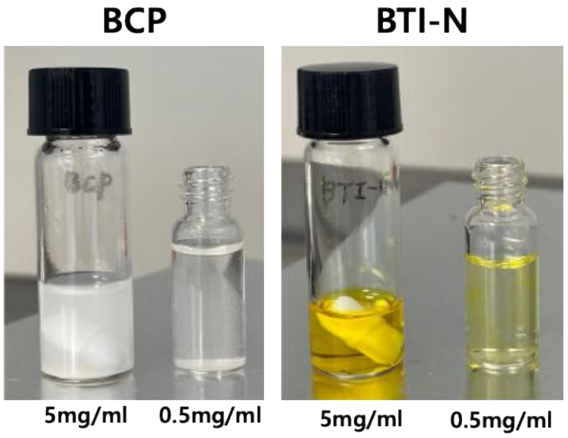

Figure S3. Solubility comparison of BCP and BTI-N in ethanol at concentrations of 0.5 mg/mL (right) and 5 mg/mL (left).

Table S2. Root mean square (R_q) and averaged roughness (R_a) values measured by Atomic force microscopy (AFM).

	PCBM-only	BCP/PCBM	BTI-N/PCBM
R_q (nm)	11.67	9.42	8.39
R_a (nm)	8.96	7.28	6.62

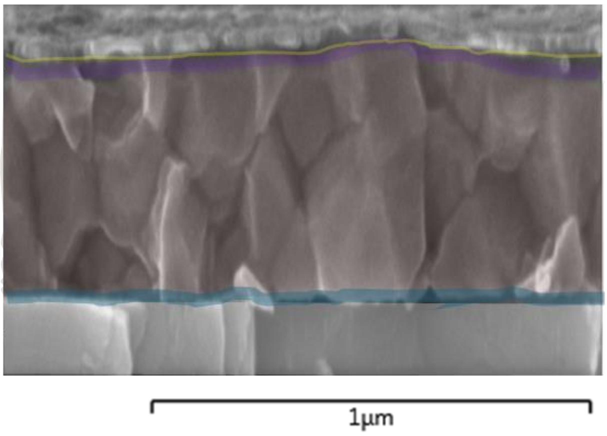

Figure S4. Scanning electron microscopy (SEM) image of solar cell device.

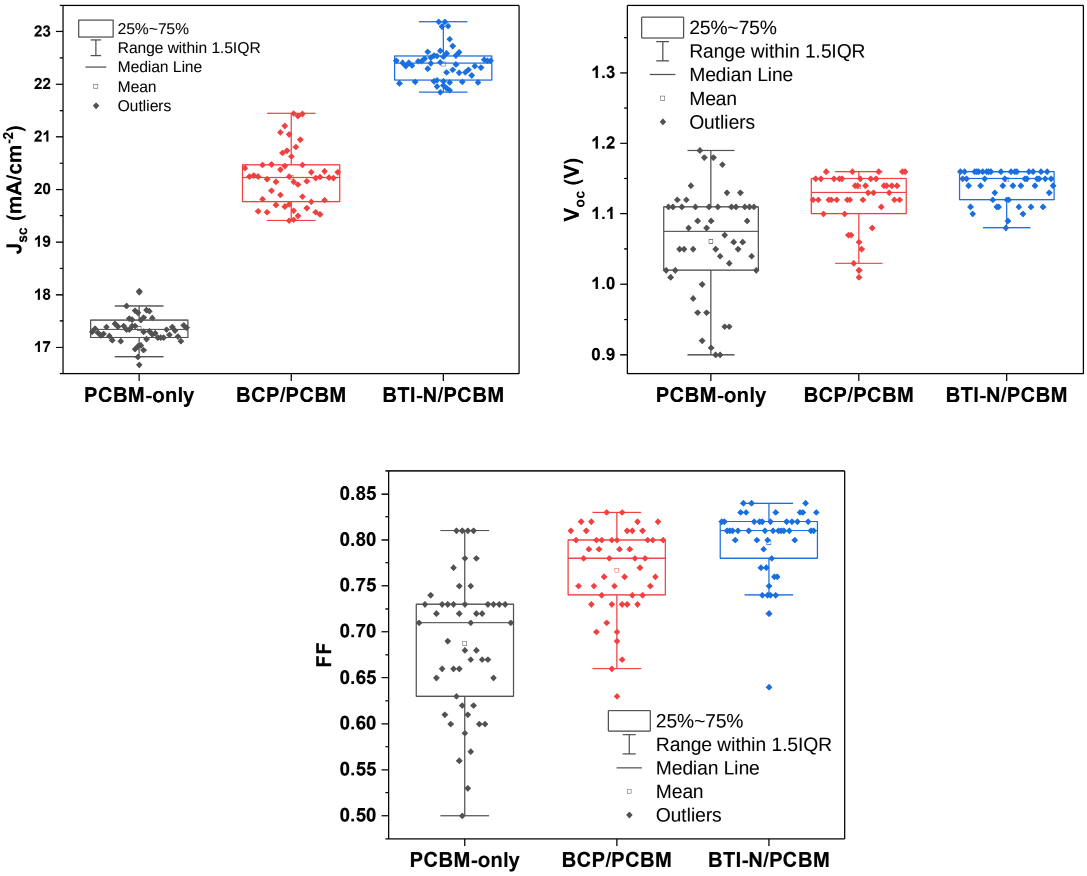

Figure S5. Statistical distribution of J_{sc}, V_{oc}, FF for PCBM-only, BCP/PCBM, and BTI-N/PCBM-based devices.

(a)

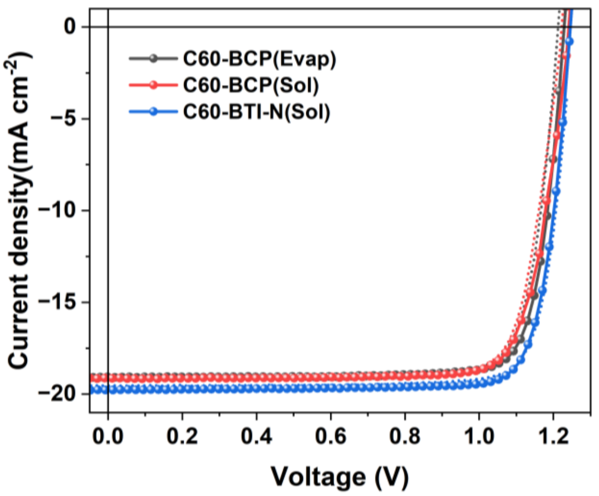

(b)

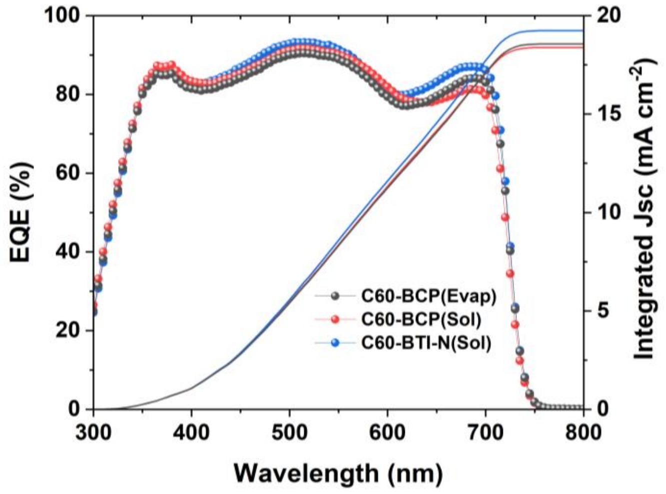

Figure S6. (a) *J*–*V* characteristics of devices for C_{60}-BCP(Evaporation), C_{60}-BCP(Solution), and C_{60}-BTI-N(Solution) based WBG perovskite devices. Solid lines represent forward scans, and dashed lines represent reverse scans. (b) EQE spectra of WBG devices.

Table S3. J-V characteristics of for C60-BCP(Evaporation), C60-BCP(Solution), and C60-BTI-N(Solution) based WBG perovskite devices under AM1.5G (100 mW/cm^2).

	J_{SC} (mA/cm^2)	V_{OC} (V)	FF	PCE (%)
C60-BCP(Evap)	19.18 (18.99 ± 0.34)	1.23 (1.20 ± 0.02)	0.82 (0.80 ± 0.03)	19.34 (18.34 ± 0.73)
C60-BCP(Sol)	19.16 (18.70 ± 0.28)	1.24 (1.21 ± 0.02)	0.79 (0.76 ± 0.05)	18.77 (17.80 ± 0.68)
C60-BTI-N(Sol)	19.90 (19.33 ± 0.19)	1.24 (1.22 ± 0.01)	0.83 (0.82 ± 0.01)	20.48 (19.43 ± 0.42)

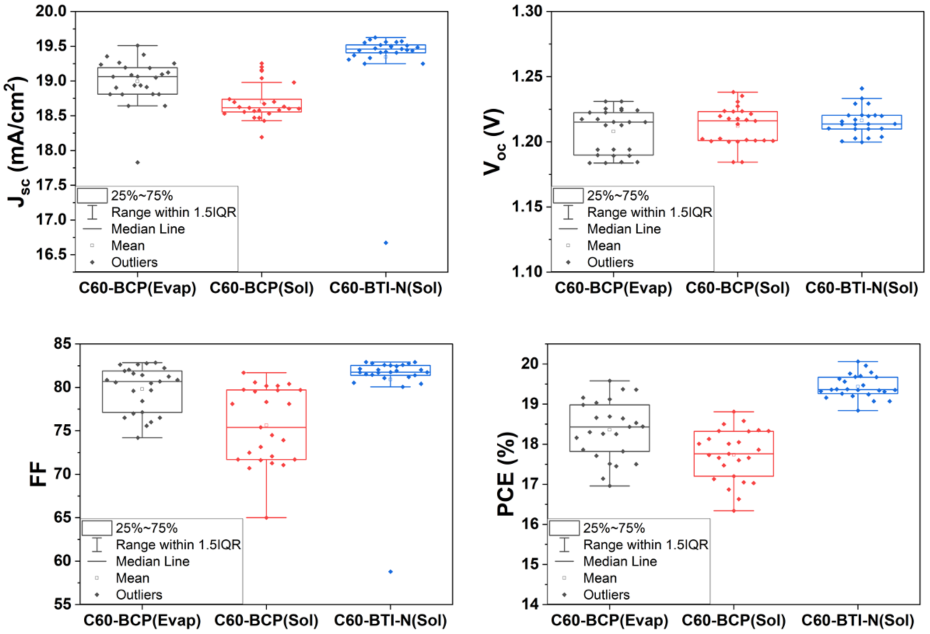

Figure S7. Statistical distribution of J_{SC}, V_{OC}, FF, PCE for C60-BCP(Evaporation), C60-BCP(solution), and C60-BTI-N(solution) based WBG perovskite devices.

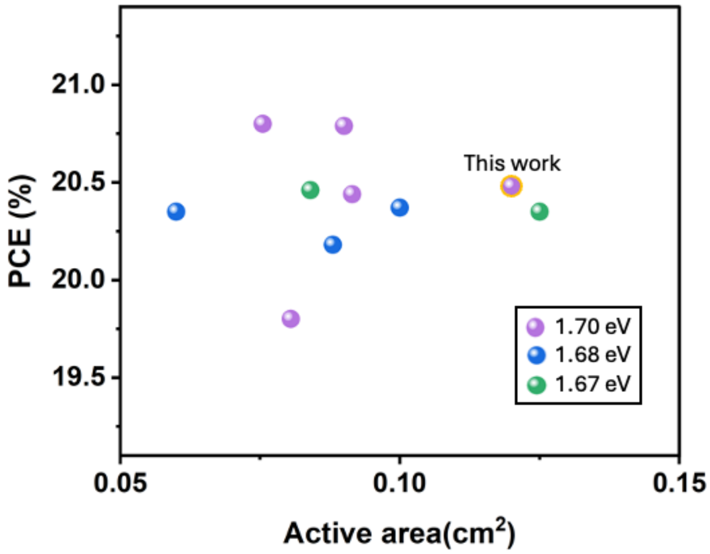

Figure S8. Competitive PCE among reported 1.67–1.70 eV systems.

Table S4. Competitive PCE among reported 1.67–1.70 eV systems.

Band gap	Active area	V_{oc} (V)	J_{sc} (mA/cm^2)	FF	PCE (%)	Reference
1.7	0.12	1.24	19.9	0.83	20.48	This work
1.7	0.0755	1.25	19.62	0.85	20.8	Wang, S.; Qi, S.; Sun, H.; Wang, P.; Zhao, Y.; Zhang, X. Nanoscale Local Contacts Enable Inverted Inorganic Perovskite Solar Cells with 20.8% Efficiency. *Angew. Chem., Int. Ed.* **2024**, 63, 202400018.
1.7	0.0805	1.30	21.1	0.73	19.8	Li, D.; Sun, X.; Zhang, Y.; Guan, Z.; Yue, Y.; Wang, Q.; Zhao, L.; Liu, F.; Wei, J.; Li, H. Uniaxial-Oriented Perovskite Films with Controllable Orientation. *Adv. Sci.* **2024**, 11, 2401184.
1.7	0.0915	1.19	20.64	0.83	20.44	Zhang, X.; Ma, Q.; Wang, Y.; Zheng, J.; Liu, Q.; Liu, L.; Yang, P.; He, W.; Cao, Y.; Duan, W.; Ding, K.; Mai, Y. Ligand Homogenized Br–I Wide-Bandgap Perovskites for Efficient NiOx-Based Inverted Semitransparent and Tandem Solar Cells. *ACS Nano* **2024**, 18, 15991–16001.
1.68	0.1	1.16	21.09	0.83	20.37	Liu, Z.; Xiong, Z.; Yang, S.; Fan, K.; Jiang, L.; Mao, Y.; Qin, C.; Li, S.; Qiu, L.; Zhang, J.; Lin, F. R.; Fei, L.; Hua, Y.; Yao, J.; Yu, C.; Zhou, J.; Chen, Y.; Zhang, H.; Huang, H.; Jen, A. K.-Y.; Yao, K. Strained Heterojunction Enables High-Performance, Fully Textured Perovskite/Silicon Tandem Solar Cells. *Joule* **2024**, 8, 2834.
1.68	0.06	1.18	21.91	0.79	20.35	Song, Z.; Sun, K.; Meng, Y.; Zhu, Z.; Wang, Y.; Zhang, W.; Bai, Y.; Lu, X.; Tian, R.; Liu, C.; Ge, Z. Universal Approach for Managing Iodine Migration in Inverted Single-Junction and Tandem Perovskite Solar Cells. *Adv. Mater.* **2025**, 37, 2410779.
1.68	0.088	1.21	20.66	0.81	20.18	Shen, J.; Li, N.; Wang, Y.; Ge, X.; Tao, J.; Yin, S.; Ning, X.; He, T.; Fu, G.; Yang, S. Delaying Crystallization and Anchoring the Grain Boundary Defects via π–π Stacked Molecules for Efficient and Stable Wide-Bandgap Perovskite Solar Cells. *Chem. Eng. J.* **2024**, 489, 151459.
1.67	0.09	1.17	20.87	0.85	20.79	Ma, Q.; Ma, M.; Liu, L.; Yang, P.; He, W.; Zhang, X.; Zheng, J.; Zhang, C.; Liu, C.; Wu, S.; Wang, Y.; Mai, Y. Wide-Band-Gap Perovskite Solar Minimodules Exceeding 43% Efficiency under Indoor Light Illumination. *Device* **2023**, 1, 100174.
1.67	0.084	1.23	20.57	0.81	20.46	Hou, F.; Guo, H.; Yang, H.; Ren, X.; Ning, X.; Li, T. Dual Interface Strategies Enable Efficient Wide-Bandgap Perovskite Solar Cells. *Appl. Phys. Lett.* **2024**, 124, 103903.
1.67	0.125	1.14	22.06	0.81	20.34	Afshord, A. Z.; Uzuner, B. E.; Soltanpoor, W.; Sedani, S. H.; Aernouts, T.; Gunbas, G.; Kuang, Y.; Yerci, S. Efficient and Stable Inverted Wide-Bandgap Perovskite Solar Cells and Modules Enabled by Hybrid Evaporation-Solution Method. *Adv. Funct. Mater.* **2023**, 33, 2301695.

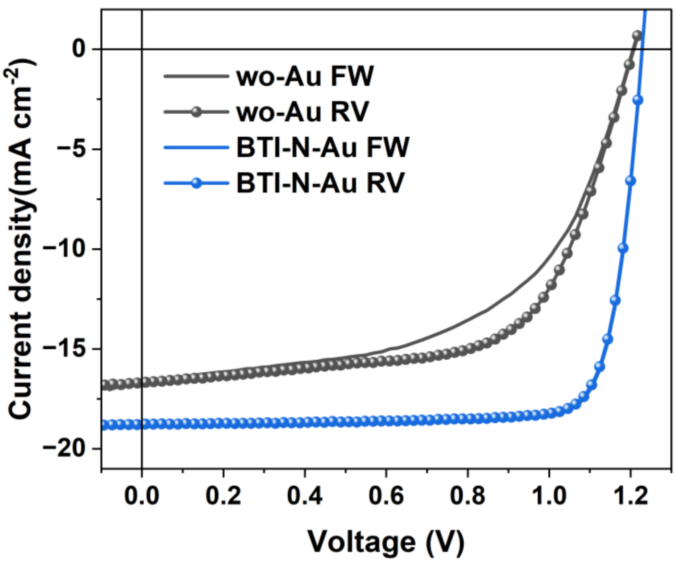

Figure S9. J–V characteristics of devices for BTI-N based WBG perovskite devices with Au.

	J_{sc} (mA/cm^2)	V_{oc} (V)	FF	PCE (%)
wo-Au	16.70<br>(16.65 ± 0.15)	1.21<br>(1.20 ± 0.02)	0.63<br>(0.57 ± 0.04)	12.73<br>(11.73 ± 0.64)
BTI-N-Au	19.04<br>(18.72 ± 0.13)	1.23<br>(1.23 ± 0.01)	0.82<br>(0.81 ± 0.01)	19.04<br>(18.57 ± 0.33)

Table S5. J-V characteristics of C60-Au and C60-BTI-N-Au WBG devices under AM1.5G (100 mW/cm^2).

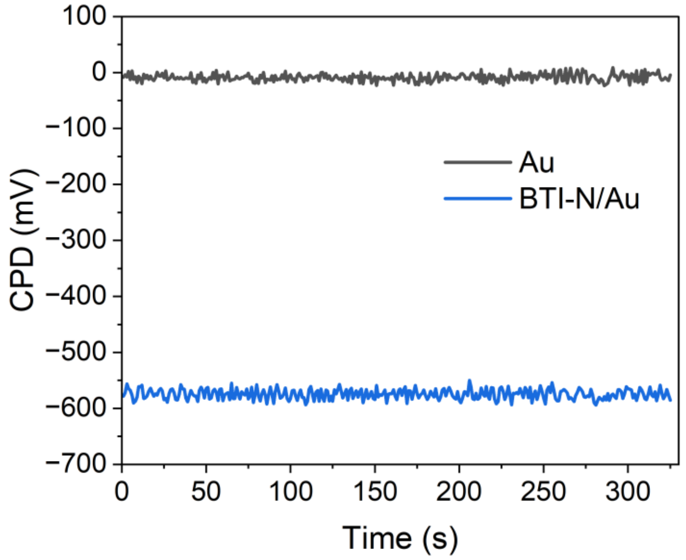

Figure S10. Kelvin probe measurements of Au and BTI-N/Au for WF

(a)

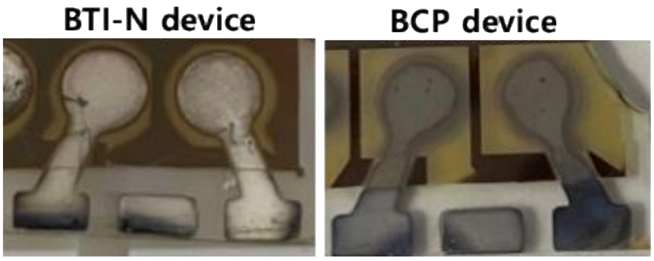

(b)

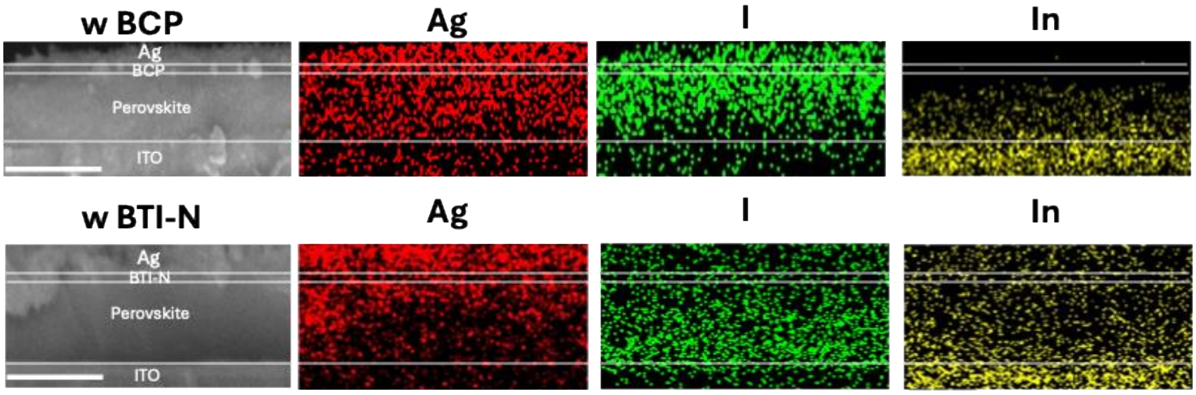

Figure S11. (a) Optical images of degraded perovskite actual devices (after 24 h at 120 °C in ambient atmosphere), (b) Cross-sectional SEM and EDS elemental mapping of Ag (red), I (green), and In (yellow) signals for thermally aged devices. The scale bar is equal to 500 nm.

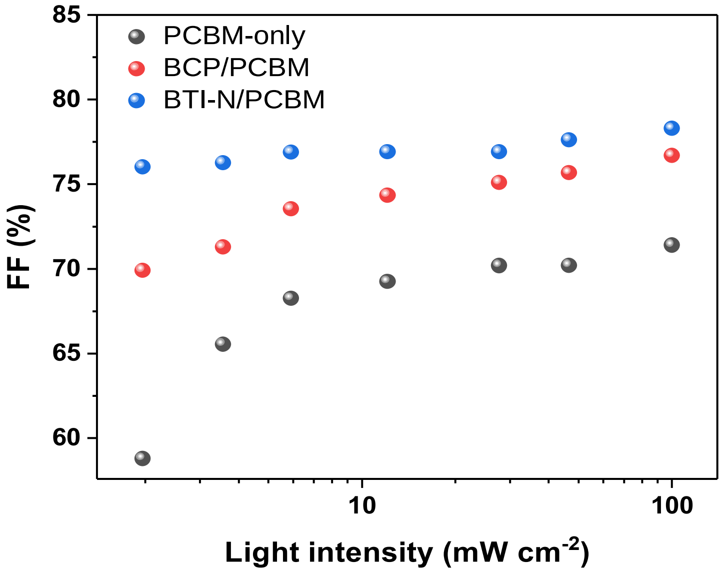

Figure S12. Light-intensity dependence of FF of devices with PCBM, BCP/PCBM, and BTI-N/PCBM.

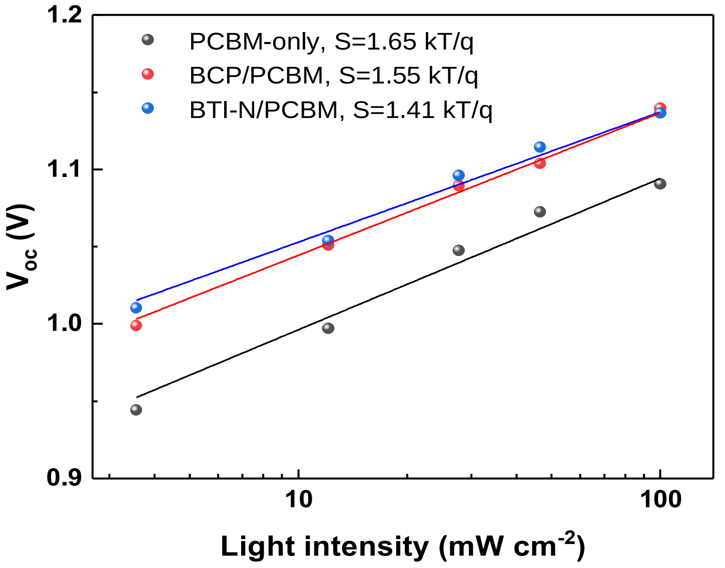

Figure S13. Light-intensity dependence of V_{oc} for with PCBM, BCP/PCBM, and BTI-N/PCBM.

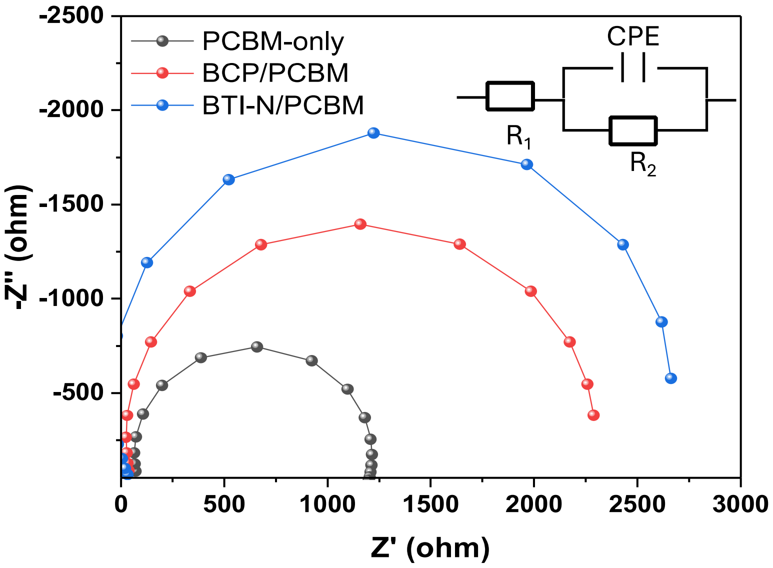

Figure S14. Nyquist plots from EIS measurements of devices with PCBM, BCP/PCBM, and BTI-N/PCBM layers.

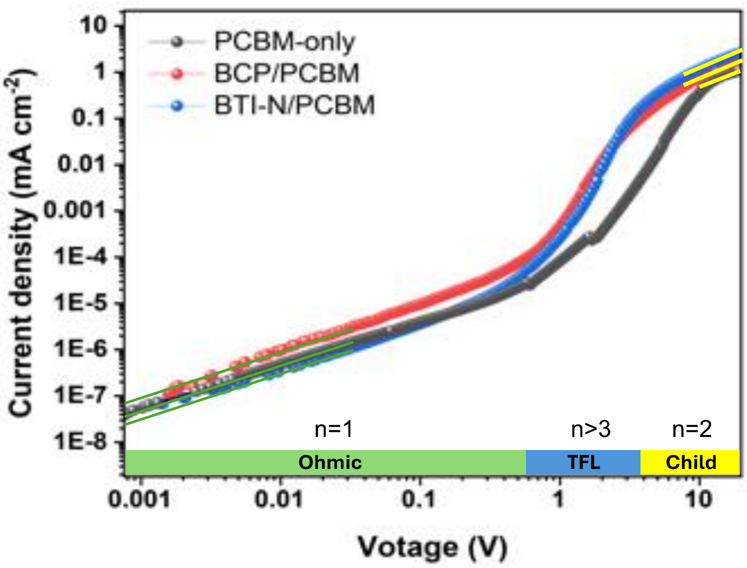

Figure S15. Space charge limited current (SCLC) on electron-only device for devices with different buffer layers under dark conditions.

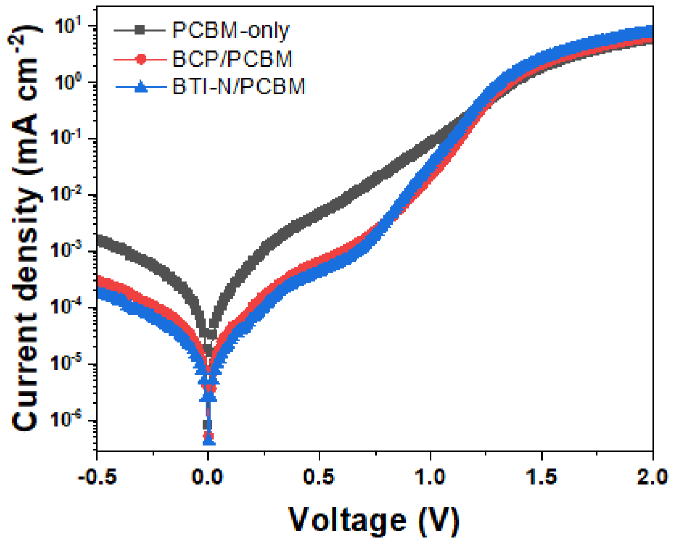

Figure S16. The dark *J–V* characteristic curves of PCBM-only, BCP/PCBM and BTI-N/PCBM device.

Table S6. Resistance, mobility and trap states density of PSCs.

	R1 (Ohm)	R2 (Ohm)	Electron mobility ($cm^2 V^{-1}s^{-1}$)	Trap states density (cm^{-3})
WO	90	1100	1.79E-06	3.22E+15
BCP	55	2210	1.02E-05	1.32E+15
BTI-N	58	2500	5.12E-05	7.29E+14
